# Corrosive Substance Ingestion: When to Perform Endoscopy?

**DOI:** 10.1111/jpc.70064

**Published:** 2025-04-21

**Authors:** Ufuk Ateş, Gülnur Göllü, Ergun Ergün, Fırat Serttürk, Anar Jafarov, Merve Bülbül, Ege Evin, Sümeyye Sözduyar, Meltem Bingöl Koloğlu, Ahmet Murat Çakmak

**Affiliations:** ^1^ Faculty of Medicine, Department of Pediatric Surgery Ankara University Ankara Turkey; ^2^ Department of Pediatric Surgery Ankara Etlik City Hospital Ankara Turkey; ^3^ Bonum Medical Klinika Sheki Azerbaijan; ^4^ Faculty of Medicine, Department of Pediatric Surgery Ege University İzmir Turkey

**Keywords:** caustics, child, dilatation, endoscopy

## Abstract

**Aim:**

Ingestion of corrosive substances is a difficult and important medical problem to manage. The aim of this study is to present an algorithm that will regulate the treatment and follow‐up of paediatric patients with corrosive ingestion.

**Methods:**

Children who were admitted to the paediatric emergency department with corrosive substance ingestions between July 2015 and December 2021 were included. Between July 2015 and January 2020, endoscopy was performed on all patients. After January 2020, endoscopy was performed only for patients presenting with hypersalivation and dysphagia.

**Results:**

172 patients were followed up and treated in our clinic due to corrosive substance ingestion. Endoscopic evaluation of 19 patients with hypersalivation revealed oesophageal corrosion stages as follows: grade I in 5 patients, grade II‐A in 1 patient, grade II‐B in 3 patients, and normal findings in the remaining patients. Of the 14 patients with dysphagia, 1 had grade I, 2 had grade II‐A, and 4 had grade II‐B corrosion, and the remaining patients had normal endoscopic findings. In the long‐term follow‐up of the patients without hypersalivation and dysphagia, no complications developed and no additional treatment was required.

**Conclusions:**

In patients presenting with suspicion of ingestion of high risk corrosive substances, there is no need for endoscopy unless hypersalivation and dysphagia are present simultaneously.

## 1 Introduction

Accidental ingestion of corrosive substances is a difficult and important medical problem to manage. Corrosive ingestion may not cause any issues, but it may also cause major problems that have a lifelong impact on the patient. Children constitute the majority of cases of corrosive ingestion, with a rate of nearly 80% [1, 2]. While corrosive ingestion develops as a result of accidents in younger children, especially infants, it also develops as a result of substance abuse in adolescents and adults [3].

In the paediatric patient population, corrosive ingestion remains a difficult situation to manage because of the unclear relationship of symptoms and signs to oesophageal injury. Although there are series in the literature in which symptoms are not effective in determining oesophageal damage, there are also series showing that the degree of damage and future strictures are always associated with symptoms and signs [4, 5]. Crain et al. stated that the association of two or more symptoms is decisive in predicting oesophageal damage [6].

Upper gastrointestinal system endoscopy is recommended in patients who swallow corrosive substances in order to reveal the damage, plan treatment and long‐term follow‐up, and shorten the hospitalisation time [7]. It is controversial to try to predict oesophageal damage due to corrosive ingestion with symptoms and signs, and to evaluate it with endoscopy [1].

The aim of this study is to present an algorithm that will regulate the treatment and follow‐up of paediatric patients presenting with corrosive ingestion through a patient‐based evaluation.

## 2 Methods

Between July 1, 2015, and December 31, 2021, 172 patients under the age of 18 who presented to the Paediatric Emergency Department with the complaint of corrosive substance ingestion were included in the study. Informed consent was obtained from all legal guardians and/or children. Approval for the study was granted by the local ethics committee (İ05‐272‐22).

Age, gender, type of corrosive substance, symptoms, physical examination findings, time of endoscopy, endoscopy findings, postoperative feeding time, medical treatments, 1‐year follow‐up findings and additional surgical intervention or dilatation data were collected.

All endoscopies were performed under general anaesthesia in operating room conditions. As shown in Table 1, the endoscopic findings of patients who had endoscopies were categorised using Zargar's grading system. All patients were called for review at the 3rd week after corrosive ingestion, during which their findings, additional examinations, and additional surgical interventions or dilatation needs were evaluated.

**TABLE 1:** Endoscopic classification of corrosive injuries according to Zargar et al. [8, 9].

Zargar classification	Description	Endoscopic view
Grade 0	Normal mucosa	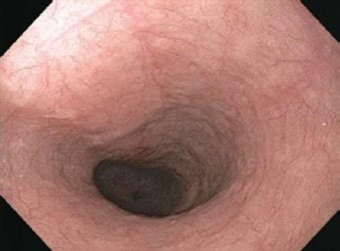
Grade I	Edema and erythema of the mucosa	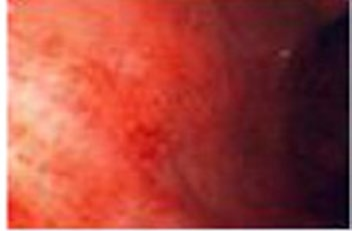
Grade II‐A	Haemorrhage, erosions, blisters, superficial ulcers	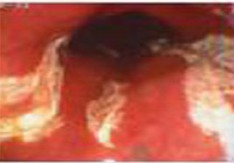
Grade II‐B	Circumferential lesions	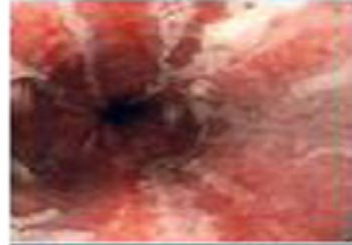
Grade III‐A	Focal deep grey or brownish‐black ulcers	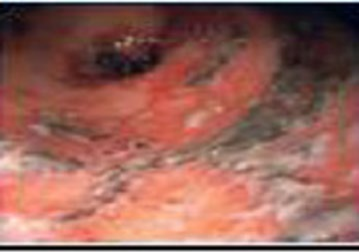
Grade III‐B	Extensive deep grey or brownish‐black ulcers	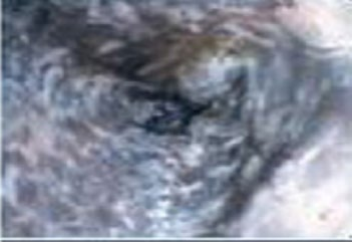
Grade IV	Perforation	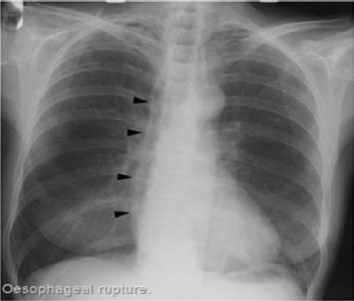

Patients' data who presented to our clinic between July 2015 and January 2020 was analysed, and a follow‐up algorithm was created by the authors. Patients who presented after January 2020 were evaluated by the algorithm, and their follow‐up was conducted accordingly.

Descriptive statistics were presented as mean, standard deviation, or median (minimum‐maximum) for quantitative variables and frequency (percent) for qualitative variables. The distribution between endoscopy status, symptom status and physical examination finding status was evaluated by Chi‐Square analysis. A univariate logistic regression analysis was used to determine the risk factors affecting the endoscopy status. The statistical significance level was accepted as 0.05, and Statistical Package for Social Sciences (SPSS, Version 15.0, Chicago, IL) was used for the analysis.

## 3 Results

Between July 1, 2015, and December 31, 2021, there were 172 patients who were followed up and treated in our clinic due to corrosive substance ingestion; 94 patients were followed up prior to the establishment of the algorithm, while 78 patients were treated according to the algorithm (after January 2020). The patient characteristics were described in Table 2. 127 patients were suspected of ingesting corrosive substances, while 45 patients were witnessed ingesting corrosive substances. Patients suspected of corrosive ingestion were often admitted to the hospital by parents with the container lid opened in the child's hand or around the child, often spilled on the patient, or with the contents contaminated in the peroral area. When ingestion was definitely witnessed, the parents saw the children swallowing or putting the contents into their mouths. We performed endoscopy on a total of 117 patients, and 23 of them underwent endoscopy according to the algorithm we developed after January 2020.

**TABLE 2:** Patient characteristics.

Age (median)	36.05 months (1–209)	
Hospital admission time after corrosive ingestion (median)	150.98 min (15–2160)	
Corrosives	Sodium Hypochloride	41 (%23.8)
	Dishwasher rinse aid	22 (%12.8)
	Lipid Remover	16 (%9.3)
	Dishwashing Liquid	13 (%7.6)
	Benzyl Salicylate	11 (%6.4)
	Nitric Acid	10 (%5.8)
	Limescale Remover	8 (%4.7)
	Washing Powder	2 (%1.2)
	Other	27 (%15.7)
	Unspecified	22 (%12.7)
Symptoms	Vomiting	55 (%32)
	Hypersalivation	31 (%18)
	Dysphagia	21 (% 12.2)
	Pain	13 (% 7.6)
	Fever	1 (% 0.6)
	Hematemesis	3 (% 1.7)
	Dyspnea	0 (%0)
	None	48 (%27.9)

There was a complaint of vomiting in 55 patients (%32). Vomiting was induced in 16 of the patients. The most common complaints among patients admitted to the hospital were vomiting and hypersalivation (Table 2). Examination findings such as redness and swelling in and around the mouth, coughing, and burns on the body were present in 36 patients. A total of 48 patients were observed to be totally asymptomatic, with 32 of them in the post‐algorithm period and 16 in the pre‐algorithm period. No cross‐sectional imaging was performed in the patients, except for chest radiography, which was utilised to rule out possible perforation and pneumomediastinum (Table 1).

**FIGURE 1:**
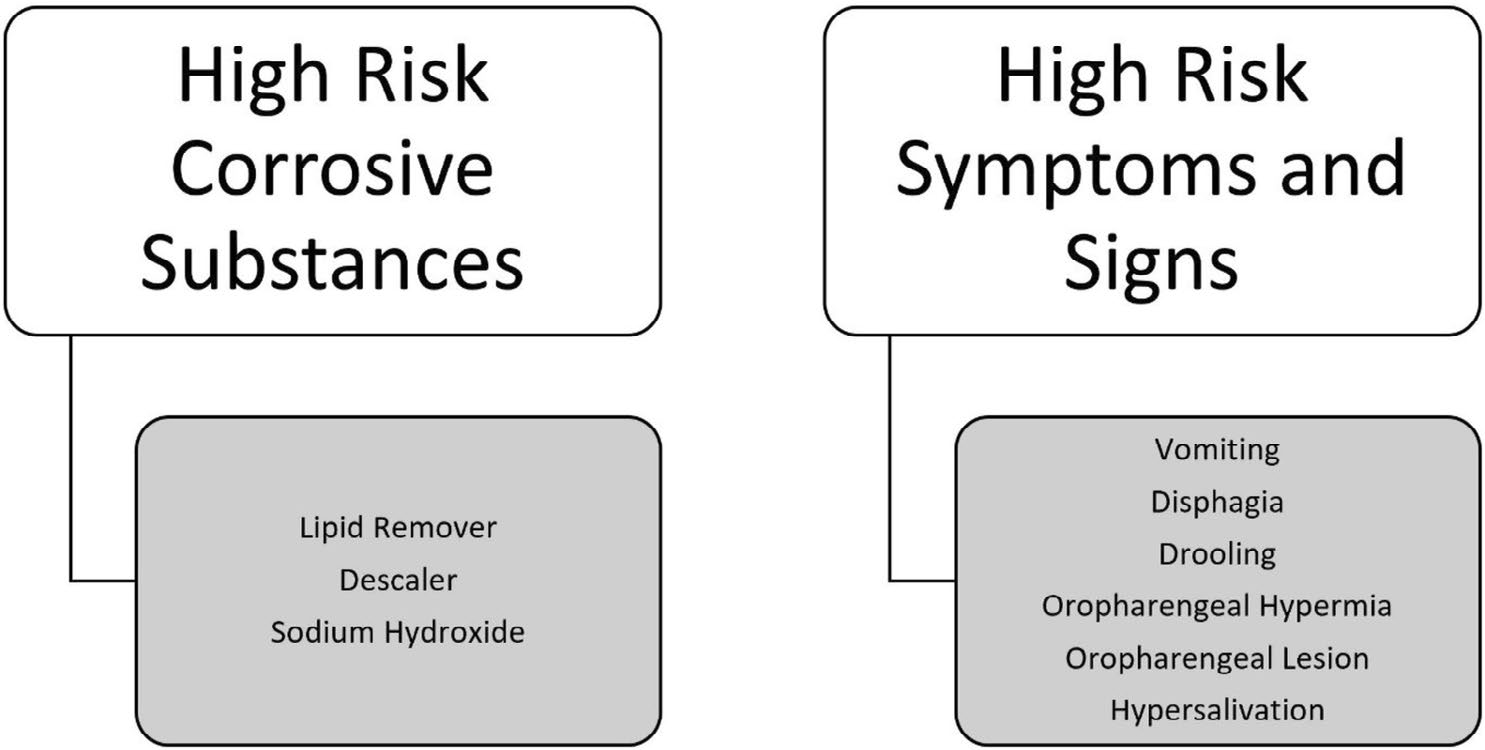
The algorithm for patients admitted to the emergency department with suspected corrosive substance ingestion.

Corrosive contents, along with symptoms and signs associated with severe oesophageal damage, were evaluated to determine the necessity of endoscopy (Figure 1). The relationship between the symptoms and physical examination findings at the time of admission to the hospital and the endoscopy grades of the patients who presented to our clinic with corrosive substance ingestion and underwent endoscopy until January 2020 was evaluated statistically.

When hospital admission symptoms and endoscopy findings were correlated, a significant correlation was observed between vomiting, inability to swallow saliva (hypersalivation), dysphagia, and pain symptoms and endoscopy findings. Table 3 presents data categorised by Zargar's Grading Classification for various clinical symptoms or findings related to corrosive ingestion with *p* values to indicate statistical significance.

**TABLE 3:** Correlation of symptoms, examination findings and endoscopy findings before algorithm.

		Zargar's grading classification				*p*
		Grade 0	Grade I	Grade II‐A	Grade II‐B	
Vomiting	Yes	15 (%60)	4 (%16)	1 (%4)	5 (%20)	0.032*
	No	55 (%79.7)	11 (%15.9)	1 (%1.4)	2 (%2.9)	
Hypersalivation	Yes	12 (%57.1)	3 (%14.3)	1 (%4.8)	5 (%23.8)	0.008*
	No	58 (%79.5)	12 (%16.4)	1 (%1,4)	2 (%2.7)	
Dysphagia	Yes	9 (%56.3)	2 (%12.5)	1 (%6.3)	4 (%25)	0.032*
	No	61 (%78.2)	13 (%16.7)	1 (%1.3)	3 (%3.8)	
Pain	Yes	3 (%42.9)	1 (%14.3)	1 (%14.3)	2 (%28.6)	0.027*
	No	67 (%77)	14 (%16.1)	1 (%1.1)	5 (%5.7)	
Hematemesis	Yes	0 (%0)	1 (%50)	0 (%0)	1 (%50)	0.095
	No	70 (%76.1)	14 (%15.2)	2 (%2.2)	6 (%6.5)	
Oropharyngeal hyperemia	Yes	38 (%77.6)	8 (%16.3)	1 (%2)	2 (%4.1)	0.736
	No	32 (%71.1)	7 (%15.6)	1 (%2.2)	5 (%11.1)	
Oropharyngeal lesion	Yes	2 (%50)	0 (%0)	0 (%0)	2 (%50)	0.087
	No	68 (%75.6)	15 (%16.7)	2 (%2.2)	5 (%5.6)	
Respiratory sounds	No	62 (%76.5)	14 (%17.3)	1 (%1.2)	4 (%4.9)	

*Note*: Bold values represent statistically significant correlations between clinical symptoms and endoscopic findings according to Zargar's Grading Classification. A *p*‐value < 0.05 was considered statistically significant in all analyses. The significant associations were identified for vomiting (*p* = 0.032), hypersalivation (*p* = 0.008), dysphagia (*p* = 0.032), and pain (*p* = 0.027).

**FIGURE 2:**
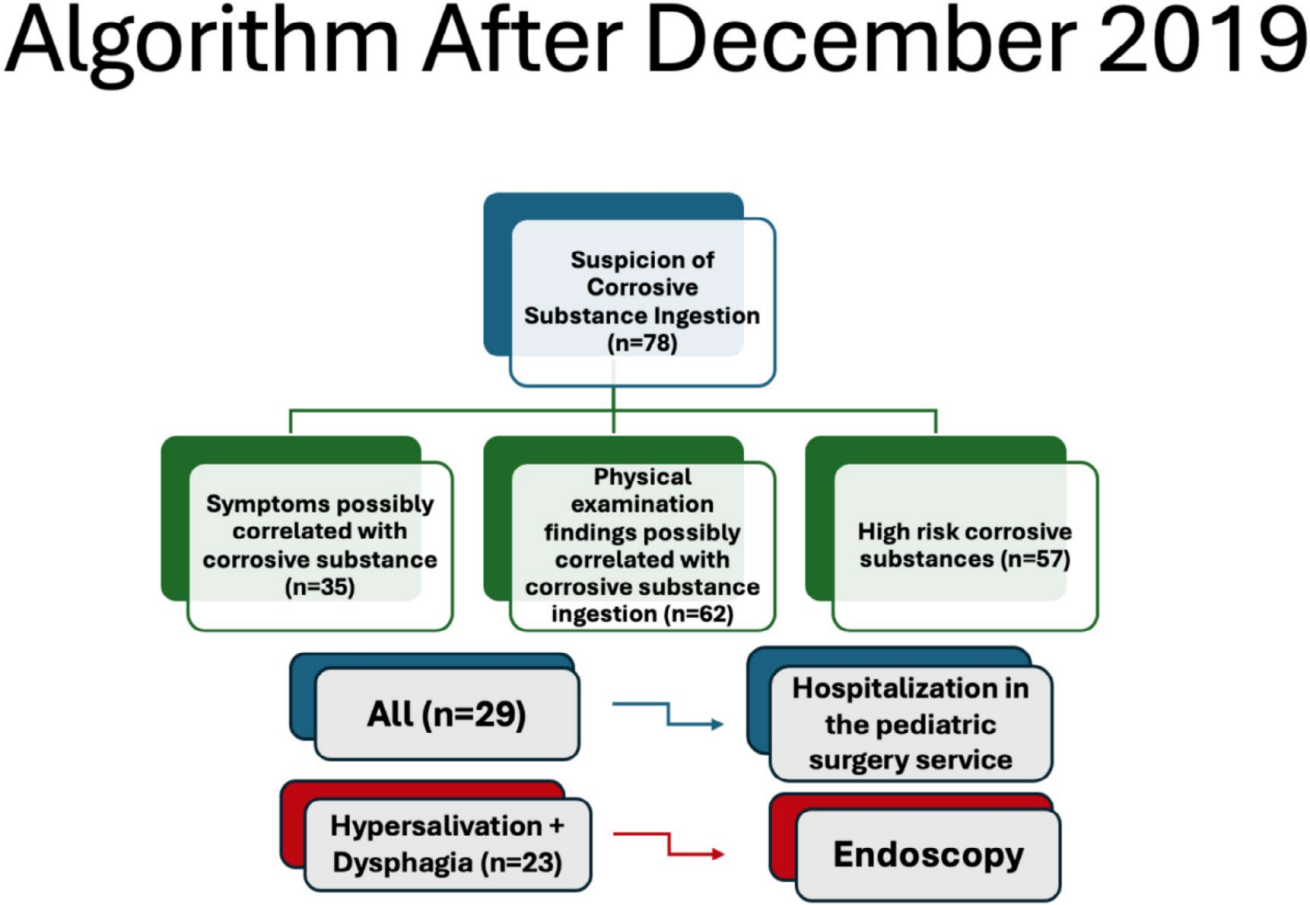
The algorithm for patients admitted to the emergency department with suspected corrosive substance ingestion. The decision to perform endoscopy was made based solely on the symptoms of hypersalivation and dysphagia.

In line with these statistics, an algorithm was created for the patients who presented to our clinic for corrosive substance ingestion. According to the established algorithm, the decision to perform endoscopy was based on symptoms. Endoscopy was performed for 23 patients in total as described in Figure 2. The algorithm was based on the statistical analysis of the previous period showing that symptoms of dysphagia and hypersalivation were statistically significantly associated with oesophageal corrosion.

As of January 2020, 78 patients who presented to our clinic due to corrosive substance ingestion were evaluated according to this algorithm. The general condition of almost all patients at admission to the hospital was good. Shock findings were not observed in any patient at the time of admission. 35 patients had symptoms possibly correlated with corrosive substance ingestion; in the physical examination of 62 patients, there were findings possibly correlated with corrosive substance ingestion, and 29 patients had both with high‐risk corrosive substance ingestion. The most common examination finding was hyperemia in the oropharynx (25%). Table 4 presents the analysis of clinical symptoms and their association with the Zargar Grading Classification on endoscopy following the introduction of the algorithm in our centre after January 2020. All 29 patients with symptoms and examination findings were hospitalised and followed up in the paediatric surgery service. The remaining 49 patients were followed in the emergency department and completed their observation period. 32 of these patients were totally asymptomatic. The patients were kept nil by mouth. Intravenous fluid, ampicillin sulbactam (100 mg/kg/g), and pantoprazole (1 mg/kg/g) treatments were administered. In the emergency department, after an observation period of 1 to 3 h, if no signs of hypersalivation or difficulty swallowing saliva were observed, patients were started on a diet of water and liquid foods. This was gradually transitioned to a normal diet. If symptoms of feeding intolerance and dysphagia were detected, it was planned to transfer the patients to the paediatric surgery service.

**TABLE 4:** Correlation of symptoms, examination findings and endoscopy findings after algorithm.

		Zargar's grading classification			
		Grade 0	Grade I	Grade II‐A	*p*
Vomiting	Yes	5 (%50)	3 (%30)	2 (%20)	0.127
	No	11 (%84.6)	2 (%15.4)	0 (%0)	
	No	15 (%78.9)	2 (%10.5)	2 (%10.5)	
Pain	Yes	1 (%25)	2 (%50)	1 (%25)	0.081
	No	15 (%78.9)	3 (%15.8)	1 (%5.3)	
Hematemesis	Yes	1 (%100)	0 (%0)	0 (%0)	0.999
	No	15 (%68.2)	5 (%22.7)	2 (%9.1)	
General situation	Middle	1 (%100)	0 (%0)	0 (%0)	0.999
	Good	15 (%68.2)	5 (%22.7)	2 (%9.1)	
Oropharyngeal hyperemia	Yes	11 (%73.3)	4 (%26.7)	0 (%0)	0.169
	No	5 (%62.5)	1 (%12.5)	2 (%25)	
Oropharyngeal lesion	Yes	2 (%66.7)	1 (%33.3)	0 (%0)	0.999
	No	13 (%68.4)	4 (%21.1)	2 (%10.5)	

Endoscopy was performed on 23 of the patients in accordance with the algorithm (Figure 2). The mean time of endoscopy was 15.2 h after corrosive ingestion (2–26 h). Findings related to corrosive substance ingestion were observed in 7 of the patients who underwent endoscopy. When these patients were evaluated according to Zargar's grading classification, 2 were grade II‐A, 5 were grade 1, and 16 were grade 0. The relationship between symptoms and corrosion levels in 23 patients who underwent endoscopy based on the newly created algorithm was re‐examined. Despite the limited sample size, we conducted a statistical analysis of the new algorithm. None of the symptoms, other than hypersalivation and dysphagia, demonstrated a significant association with corrosion levels (Table 4).

The mean postoperative oral feeding time of these patients was 12.3 h (2–96 h) Intravenous methylprednisolone (2 mg/kg/g) treatment was started in 2 patients with severe oesophageal damage detected during endoscopy, to reduce the inflammatory response that would develop during wound healing and thus prevent stricture formation. The methylprednisolone treatment was discontinued by reducing the dose. Following the endoscopy, patients who were confirmed to be complaint‐free and able to feed themselves were discharged. Six patients, whose symptoms were followed up without endoscopy, were discharged after feeding was started, as they had no complaints. Paediatric surgery outpatient review was recommended to the patients 3 weeks after discharge. None of these patients had any additional complaints during their 1‐year follow‐up.

In the endoscopy group, from the patients who returned for their third‐week follow‐up, three reported ongoing complaints. Two patients experienced difficulty swallowing, and one patient had regurgitation. A contrast study was performed on these patients, and there were no pathological findings. None of the patients in the endoscopy group who presented to the 8th week review had complaints.

49 patients (28.5%) who did not have complaints or examination findings were discharged after completing the observation period in the emergency department. After the feeding was started, they had no complaints. Paediatric surgery outpatient review was recommended to the patients 3 weeks after discharge. None of the patients had any complaints during the 1‐year follow‐up.

All patients included in the study were meticulously followed up. For those who could not attend their scheduled follow‐ups, information was obtained via phone to determine whether they had any complaints. A total of 48 patients (27.9%) were totally asymptomatic across the study. These patients were followed up for 1 year. None of the asymptomatic patients in either group (with or without endoscopy) experienced any complications, such as oesophageal strictures or delayed symptoms, during the one‐year follow‐up period. This finding supports the conclusion that withholding endoscopy in asymptomatic patients does not compromise clinical outcomes, thus validating the proposed algorithm.

## 4 Discussion

Accidental ingestion of corrosives is a preventable public health problem. It can cause burns in the oesophagus, according to the corrosive substance's properties. Corrosive ingestion is more common in children from families with a lower socioeconomic level [10]. The damage will vary depending on many components, such as pH value, concentration, physical condition, fluidity, amount of drink, and exposure time. It has been seen that the majority of the corrosive substances swallowed are products used in household cleaning, and they turn into a danger that is easy for children to reach if they are not stored under appropriate conditions [11]. In our study, the majority of the corrosive substances were substances used in household cleaning. In particular, the risk of corrosive ingestion from household cleaning products can be reduced by limiting the use of corrosive substances, utilising child‐resistant packaging, and placing clear, easy‐to‐understand warning labels on the products.

Moderate and severe oesophageal injuries are less common in asymptomatic patients. Betalli et al. grouped the symptoms of the patients into major and minor symptoms. Minor symptoms were defined as oropharyngeal lesions and vomiting; major symptoms were defined as dyspnea, dysphagia, drooling and hematemesis.

Some authors do not consider it necessary to perform endoscopy in patients without oropharyngeal lesions [7, 12, 13, 14, 15]. Signs of developing oesophageal damage include oropharyngeal pain, chest pain and dysphagia. Accompanying symptoms such as vomiting, hypersalivation and stridor may be related to the severity of the damage [16]. Urganci et al. reported that no pathological finding was observed in 53% of the patients evaluated by endoscopy [17]. Gupta et al. also reported no pathological findings in the endoscopic examination performed on asymptomatic patients, and they detected advanced injuries only in patients with severe symptoms [18]. In light of the findings obtained in the multicentre study conducted by Di Nardo et al., they recommended that only symptomatic patients undergo endoscopy [1].

In 48 (27.9%) of the patients included in our study, no symptoms related to corrosive ingestion were identified. In patients with symptoms, the most common symptom was found to be vomiting. Vomiting in 16 of 55 patients was described as induced vomiting. Oral mucosal damage and hypersalivation are the most common symptoms in patients with injuries [17]. In our study, hypersalivation and dysphagia were the most common symptoms in the presence of oesophageal injury.

Ingestion of corrosive, acidic or alkaline substances may affect the intestinal or respiratory systems, depending on their content. Since the acid content causes coagulation necrosis and tissue penetration is weak, the possibility of damaging the surrounding tissues and developing stenosis is lower. Since the ingestion of alkaline content causes liquefaction necrosis, tissue penetration is much higher and leads to the development of potential complications more frequently [16]. It has been observed that substances with an alkaline content are more effective in the formation of strictures [1]. Some of these products, like lipid dissolver and limescale dissolver, were associated with oesophageal injury in our retrospective analysis.

Cross‐sectional imaging may be considered a sensitive tool for excluding upper gastrointestinal mucosal injuries after acute caustic ingestions. However, Bahrami‐Motlagh et al. demonstrated that the correlation between endoscopy and CT scan findings regarding injury grading is not sufficient to eliminate the need for endoscopy. In our clinic, chest radiography is utilised solely to detect possible perforation at an early stage, minimising radiation exposure and avoiding unnecessary imaging, particularly in children [19].

Bicakci et al. did not evaluate the patients with endoscopy in the early period but evaluated the patients with contrast study if the symptoms persist after 3 weeks and followed the patients with dilatation if there is a stricture [5]. They stated that the findings to be obtained by endoscopy are not a determining factor in determining the course of treatment. They stated that endoscopy should be performed only in patients with feeding difficulties after corrosive ingestion [5].

The patients in our series were evaluated in the outpatient clinic. 3 weeks after the first hospital admission, oesophageal stenosis was investigated by contrast study in the case of ongoing symptoms. In our series, stenosis developed in only one patient. The patient who swallowed sodium hydroxide had symptoms of dysphagia, hypersalivation and vomiting at the emergency service admission, and ulcerated lesions in the oropharynx were observed in the examination findings. Grade II‐A damage was observed in the endoscopy. In another patient, there was hematemesis as an ongoing symptom at the 3rd week's evaluation. No stenosis was observed in the contrast study, but an ulcerated lesion was observed in the upper part of the oesophagus in the control endoscopy (also seen in the first endoscopy). The patient's complaints completely disappeared in the follow‐up.

The retrospective nature of the study is one of the main limitations. The authors think that better results may be obtained with randomised studies with a larger number of patients. Another pitfall of the study that should be mentioned is that the patients who had undergone endoscopic dilatation or other procedures due to corrosive substance ingestion in other centres were excluded from the study since the management of these children varies between centres.

## 5 Conclusions

Corrosive ingestion and related potential pathologies are conditions that maintain their severity. It has been shown that the ingested substance, symptoms and signs can give us preliminary information about damage. It has been observed that even findings such as drooling, hypersalivation and dysphagia can be indicators of damage. The role of endoscopy is important in planning the probability of developing stenosis and treatment management in the long term. However, it is not considered necessary to perform endoscopy on every patient. The authors recommend endoscopic evaluation of patients in the presence of dysphagia, inability to swallow, and hypersalivation regardless of corrosive content.

## Ethics Statement

Informed consent was obtained from all legal guardians and/or children. Approval for the study was granted by the local ethics committee (İ05‐272‐22).

## Conflicts of Interest

All authors (Ufuk Ates, Gulnur Göllu, Ergun Ergun, Fırat Sertturk, Anar Jafarov, Merve Bulbul, Ege Evin, Sumeyye Sozduyar, Meltem Bingol Kologlu, Ahmet Murat Cakmak) have contributed significantly, they all are in agreement with the content of the manuscript. There is no conflicts of interest and no external funding for this study. All authors contributed significantly to the study according to the guidelines of International Committee of Medical Journal Editors (ICMJE).
